# DM-YOLO: improved YOLOv9 model for tomato leaf disease detection

**DOI:** 10.3389/fpls.2024.1473928

**Published:** 2025-02-11

**Authors:** Abudukelimu Abulizi, Junxiang Ye, Halidanmu Abudukelimu, Wenqiang Guo

**Affiliations:** School of Information Management, Xinjiang University of Finance and Economics, Urumqi, China

**Keywords:** tomato leaf disease detection, YOLO, DM-YOLO, sampling method, loss function

## Abstract

In natural environments, tomato leaf disease detection faces many challenges, such as variations in light conditions, overlapping disease symptoms, tiny size of lesion areas, and occlusion between leaves. Therefore, an improved tomato leaf disease detection method, DM-YOLO, based on the YOLOv9 algorithm, is proposed in this paper. Specifically, firstly, lightweight dynamic up-sampling DySample is incorporated into the feature fusion backbone network to enhance the ability to extract features of small lesions and suppress the interference from the background environment; secondly, the MPDIoU loss function is used to enhance the learning of the details of overlapping lesion margins in order to improve the accuracy of localizing overlapping lesion margins. The experimental results show that the precision (P) of this model increased by 2.2%, 1.7%, 2.3%, 2%, and 2.1%compared with those of multiple mainstream improved models, respectively. When evaluated based on the tomato leaf disease dataset, the precision (P) of the model was 92.5%, and the average precision (AP) and the mean average precision (mAP) were 95.1% and 86.4%, respectively, which were 3%, 1.7%, and 1.4% higher than the P, AP, and mAP of YOLOv9, the baseline model, respectively. The proposed detection method had good detection performance and detection potential, which will provide strong support for the development of smart agriculture and disease control.

## 1 Introduction

The tomato is an annual herbaceous plant that is widely grown worldwide and is an important source of income in many agricultural countries. Owing to environmental and climatic factors, tomatoes are highly susceptible to bacterial and viral infections, which seriously affect their yield and quality. Initial symptoms of leaf diseases usually appear on the surface of leaves, and early detection and identification of the diseases are crucial to reducing mutual infection and spread among tomato plants; therefore, accurate disease identification becomes especially critical (Yao et al., 2023). Conventional disease detection mainly relies on the empirical judgments of agricultural experts, which is not only inefficient but also has poor consistency of results, making it difficult to meet the needs of modern efficient agriculture. In recent years, as computer vision (CV) and deep learning (DL) have been widely favored by the academic community, the integration of leaf disease detection technique into tomato production has become an important trend in modern tomato planting.

DL has significantly improved the performance of deep neural networks with its excellent self-directed learning capability, which has become a frontier and new trend of tomato disease detection (Sunil et al., 2023). Compared with conventional methods, DL algorithms have advantages in detection speed, detection accuracy, and generalizability (Liu and Wang, 2021b). Currently, mainstream object detection algorithms include Faster R-CNN (Ren et al., 2017), SSD (Single Shot MultiBox Detection) (Liu et al., 2016), and YOLOs (You Only Look Once) (Liu and Wang, 2021a; Li et al., 2022; Wang et al., 2023a). Based on these algorithms, researchers have conducted a number of studies on tomato disease detection, demonstrating the great potential and advantages of DL algorithms in disease detection. Under different detection environments (Zayani et al., 2024), built independently a greenhouse tomato leaf disease dataset and proposed an automated disease detection model based on YOLOv8, by which an accuracy of 66.7% was achieved. Meanwhile (Wang et al., 2021), introduced the Dense module into YOLO and increased the tomato detection accuracy to 96% while varying scale and density. On this basis (Jin et al., 2023)performed multiscale feature fusion by adding the Convolutional Block Attention Module (CBAM) and the Weighted Bidirectional Feature Pyramid Network (BiFPN), so that it became easier to deploy the algorithm on disease detection equipment, and an online disease diagnosis platform has been developed. In terms of model lightweighting (Zeng et al., 2023)reconstructed the backbone network using downsampled convolutional layers and MobileNet to lighten the model structure. Meanwhile (Umar et al., 2024)integrated the Simple Parament-Free Attention Module (SimAM), Dual Attention-in-Attention Module (DAiAM), and the Max Pooling Convolution (MPConv) structure into the YOLOv7 network architecture, which enabled model lightweighting while increasing the accuracy. However, in contrast (Albattah et al., 2021)proposed a DenseNET-77-based framework for automatic plant disease detection that could not be deployed on mobile devices due to its failure to take into account the model volume.

However, the above studies were conducted only in the greenhouse environment, yet in the actual natural detection environments, factors such as light variation, symptom overlap, and small lesion area present many challenges to tomato leaf disease detection. For example, it is difficult to localize a lesion due to light change, different diseases have similar symptoms, the detection area is small owing to leaf occlusion, etc. In order to address these challenges, related researchers have proposed methods for tomato leaf disease detection in natural environments. First (Roy et al., 2022), proposed a high-performance real-time fine-grained object detection framework based on YOLOv4, thereby such problems as dense distribution, irregular shape, and texture similarity in plant disease detection were solved. Meanwhile (Tang et al., 2023), proposed a PLPNet-based method for tomato leaf disease detection, which introduces an adaptive convolution module and a location-enhanced attention mechanism to suppress the interference from soil background. In terms of specific disease detection (Liu and Wang, 2023) introduced a hybrid attention mechanism into the feature prediction structure of YOLOv5 to improve the detection accuracy of tomato brown spot disease in complex scenes, while (Liu and Wang, 2021b) incorporated MobileNetv2 into YOLOv3 for early identification of tomato gray spot disease. In terms of leaf occlusion and overlap detection (Wang et al., 2021)proposed the YOLOv3-tiny-IRB algorithm motivated by the idea of an inverse residual block, addressing effectively the problems of light variations and tree branch occlusion. Meanwhile, by combining the improved YOLOv5 with ShuffleNet (Li et al., 2022), enabled precise detection of peach tree leaf diseases in natural environments. Although the lightweight improvement led to a slight decrease in accuracy, the detection effect was still satisfactory (Zhang et al., 2022). enabled real-time detection of cotton diseases and insect pests in complex natural environments by introducing the Efficient Channel Attention (ECA) mechanism, hard-Swish function, and Focal Loss function into YOLOX. Finally (Gao et al., 2024), introduced the Adaptive Feature Extraction Network (AFEN) and Cross-layer Feature Extraction Network (CFFN) and proposed a new LACTA algorithm, resulting in higher detection accuracy of cherry tomato diseases in an unstructured environment. In addition (Wang Y et al., 2024), proposed a tomato disease detection method incorporating CBAM and multiscale re-parameterized generalized feature fusion (BiRepGFPN) based on YOLOv6 (Liu and Wang, 2023). proposed an object detection algorithm with a prior knowledge attention mechanism and additional new feature fusion layers and prediction layers (PKAMMF)to address the challenges of dense object distribution and insufficient feature information of small objects, with an AP of 91.96% on a self-constructed tomato disease dataset (Qi et al., 2023). proposed a tomato viral disease detection method based on SE-YOLOv5, which extracts key disease features using the squeeze-excitation (SE) mechanism, resulting in higher detection accuracy (Li et al., 2022). proposed a multiscale cucumber disease detection method in natural scenes combining coordinate attention (CA) and Transformer mechanisms to reduce the interference from invalid background information. Besides, in literature (Guo et al., 2021; Zhao et al., 2022; Cai and Jiang, 2023; Li et al., 2022; Li K. et al., 2023; Guan et al., 2024; Zhang et al., 2024; Zhu et al., 2024) the detection of leaf diseases of grapes, strawberries, passion fruits, maize, wheat, olives, and other plants in natural environments was also enabled from the perspectives of multi-scale feature fusion and the attention mechanism, and in all the cases, excellent detection effects were achieved.

Although good outcomes have been achieved in the above studies in terms of tomato leaf disease detection, accurately identifying disease classes in natural environments remains a tough challenge. A rise in the false detection rate is jointly caused by factors such as light variation-induced shadows being easily confounded with the spots caused by tomato leaf mold, small detection area due to leaf occlusion, and fewer lesion features at the early stage of early blight. In view of the urgent need to improve the performance of existing tomato leaf disease detection methods to address the above problems, the authors incorporated the point sampling operation of DySample (Liu et al., 2023) and adaptively adjusted the positions and density of the sampling points, enabling more precise capture of the fine lesion features so as to improve the accuracy of disease detection and to enhance the ability to detect small lesion features; moreover, by using the MPDIoU (Ma and Xu, 2023), the model pays more attention to the marginal details of features during the training, thus enhancing the ability to learn fuzzy margin areas and improving the localization accuracy of overlapping margins of lesions, so as to effectively solve the above detection challenges.

The YOLO family of algorithms is widely favored in the field of disease detection for its delicate balance between speed and accuracy, among which YOLOv9 (Wang C. et al., 2024) performs particularly well in terms of inference speed and detection accuracy. Therefore, YOLOv9 is chosen as the baseline model in this paper. However, this model has still some limitations for such problems as light variation, small size of lesion location, and overlapping symptoms. To address these problems, this paper proposes an improved tomato leaf disease detection method based on YOLOv9, which mainly has theoretical and practical contributions as follows.

Theoretical contribution: The unique Programmable Gradient Information (PGI) and Generalized Efficient Layer Aggregation Network (GELAN) architectures of YOLOv9 are used to effectively capture tomato leaf disease information at different levels and scales and enhance the model’s ability to perceive and capture small lesion features of tomato leaf diseases, enabling effectively rapid detection of different classes of leaf diseases.

Practical contribution: With the integrated DySample and MPDIoU, more detailed and accurate fine feature information of diseases can be obtained, the marginal detail features of the lesions can be captured, and the marginal detail learning can be enhanced to identify effectively the early fine lesion areas, enabling accurate detection of tomato diseases and precise localization of the fine marginal features of the lesions at different scales.

## 2 Related work

Object detectors: The core of an object detector is to efficiently classify and localize objects of interest with low delay, which is crucial for practical applications. In recent years, researchers have invested a lot of efforts in developing efficient detectors (Zhang et al., 2022; Lu et al., 2022; Zhang et al., 2020). In particular, YOLO algorithms (Wang C. et al., 2024; Redmon et al., 2016; Redmon and Farhadi, 2017, Redmon and Farhadi, 2018; Bochkovskiy et al., 2020; Ge, 2021; Glenn, 2022; Li et al., 2023; Wang et al., 2023a; Varghese and Sambath, 2023; Wang Y. et al., 2024) have stood out from numerous detectors due to their excellent performance. Since its inception, the YOLO has evolved continuously and a number of its versions have been iteratively released. In YOLOv1 (Redmon et al., 2016), YOLOv2 (Redmon and Farhadi, 2017), and YOLOv3 (Redmon and Farhadi, 2018), a typical network architecture, i.e., backbone–neck–head, is used. In YOLOv4 (Bochkovskiy et al., 2020) and YOLOv5 (Glenn, 2022), the Cross Stage Partial Network (CSPNet) (Wang et al., 2020) is introduced in place of the original DarkNet (Wang et al., 2019) to optimize the network structure. In YOLOv6 (Li et al., 2023), the network structure is further optimized by introducing Bidirectional ConvLSTM network (BiC) and Simultaneous Cross Stage Partial Spatial Pyramid Pooling Feature (SimCSPSPPF). In YOLOv7 (Wang et al., 2023a), the E-ELAN architecture is introduced to enrich the gradient information. In YOLOv8 (Varghese and Sambath, 2023), the C2f module is proposed for feature extraction and feature fusion. Gold-YOLO (Wang et al., 2023b) enhances the multiscale fusion capability through an advanced GD mechanism. In YOLOv9 (Wang C. et al., 2024), PGI and GELAN are introduced to solve the problems of information loss and reversibility. In the latest YOLOv10 (Wang Y. et al., 2024), a dual training strategy without non-maximum suppression (NMS) and a model structure design based on accuracy-efficiency driving are introduced, enabling end-to-end real-time detection.

Disease detection: Disease detection is an integral part of the agricultural production process. With the rapid development of DL technique, disease detection algorithms have received extensive attention from the academic community. Researchers are committed to developing practical disease detection frameworks and algorithms, and have proposed various YOLO-based algorithms and their variants, which have significantly improved the performance and efficiency of disease detection. The YOLOv4-based detection framework (Aldakheel et al., 2024) was trained for disease classification on a dataset of fourteen plant leaf diseases and showed good performance. YOLO-NAS (Hicham et al., 2024) was extensively trained on a comprehensive dataset including different lights and backgrounds, making the detection more robust. In YOLOv5-CBAM-C3TR (Lv and Su, 2024), an attention mechanism and a Transformer-based module are introduced for apple leaf disease detection, making subsequent classification more convenient. In YOLOv8-Grad-CAM++ (Quach et al., 2024), a tomato fruit health inspection system with real-time tracking and counting functions is built to further improve the detection accuracy and efficiency.

## 3 Methodology

### 3.1 DM-YOLO

**Figure 1:**
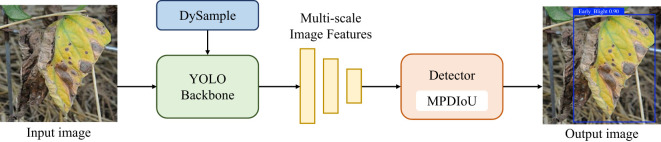
The overall workflow of DM-YOLO,including data input,feature extraction and fusion, and out of detection map at the detector.

As illustrated in **Figure 1**, the DM-YOLO framework in this paper consists primarily of three components: the head network, neck network, and detector. The head network features a series of convolutional blocks designed to extract shallow and deep features of various scales from the input image. The neck network integrates the lightweight DySample module to dynamically perform differential sampling and feature fusion on disease characteristics, forwarding these to the model’s detection head. The detection head then utilizes MPDIoU to calculate the loss between the predicted and target boxes, ultimately generating the detection map.

**Figure 2:**
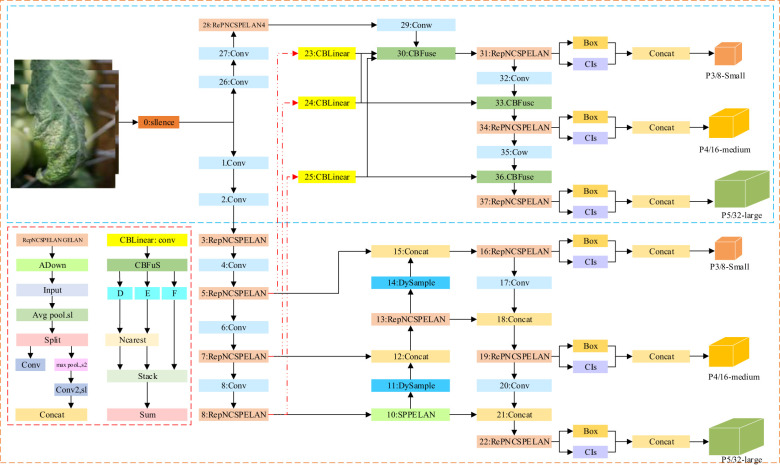
DM-YOLO network structure diagram.

For the above problems, the authors improved YOLOv9 in two key aspects to improve its tomato disease detection performance in natural environments. Firstly, a lightweight upsampler, DySample, was integrated into the backbone network, enabling finer collection of image samples with similar symptoms by automatically adjusting the sampling strategy so as to efficiently extract small lesion features, suppress the interference from invalid information, and accurately identify similar diseases. Secondly, a new loss function, MPDIoU, was used, which not only strengthened the model’s ability to learn details of overlapping margins but also further improved the ability to accurately locate and differentiate the overlapping lesion margins, helping accurate localization of overlapping areas. The improved DM-YOLO architecture is shown in **Figure 2**.

#### 3.1.1 DySample

**Figure 3:**
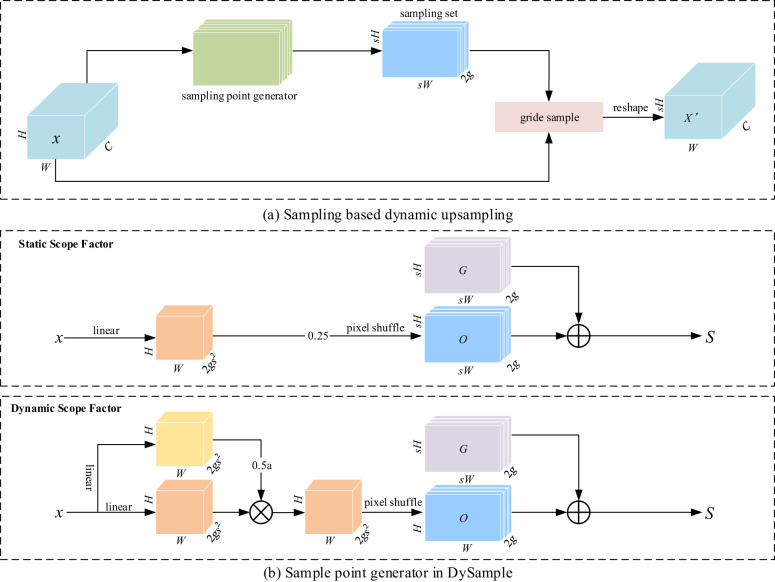
**(A, B)** DySample network structure diagram.

The YOLOv9 (Wang C. et al., 2024) algorithm is not so sensitive to the information of images with similar disease features during image sampling, failing to differentiate images with similar symptoms. To solve this problem, DySample (Liu et al., 2023), an efficient sampler, was introduced, which improved the sampling efficiency for similar disease images and suppressed unwanted background information by automatically learning different features. Its detailed framework is shown in **Figure 3**. DySample combines the initial sampling position and offset and captures the disease features more accurately by dynamically adjusting the sampling point, resulting in higher detection accuracy. Its implementation is detailed as follows:

Return to the essence of upsampling, i.e., point sampling: The feasibility of sampling-based dynamic upsampling design was demonstrated using PyTorch built-in functions, as shown in **Figures 3A, B**.

Control the initial sampling position: Given two feature mappings: source feature mapping *X* of size $C\times H_{1}\times W_{1}$ and object sampling set *S* of size $2\times H_{2}\times W_{2}$ . A grid sample function was used to resample the hypothetical bilinear interpolation *X* into $X^{'}$ of size $C\times H_{2}\times W_{2}$ , as shown in Equation 1:


(1) $$X^{'}=gride\_sample(x,S),$$

Adjust the offset moving range: Given an upsampling scale factor *S* and a feature mapping *x* of size C×H×W , an offset *O* of size 2s×H×W was generated via a linear layer with input and output being *C* and $2s^{2}$ , respectively, and finally, the offset was reshaped by pixel shuffle into 2×sH×sW . In order to constrain the local offset range, a “static scope factor was introduced, and the offset was multiplied by 0.25 to satisfy the boundary condition between overlapping and non-overlapping lesion margins. This process is illustrated in Equation 2–Equation 4.


(2) O=linear(X),

(3) S=G+O,

(4) O=0.25linear(x).

Introduction of dynamic scope factors: The introduction of dynamic factors enables the model to handle various complex features more accurately. Point-by-point “dynamic scope factors were generated by linear projection, and the use of a sigmoid function with a dynamic factor of 0.5 ensures flexible adjustment of sampling under different features and environments, so that the offset of each point is not only subject to the static factor but also adjustable depending on the dynamic factor, as detailed in Equation 5.


(5) $$O=0.5sigmoid(linear_{1}(x))•linear_{2}(x).$$

The dynamic upsampling mechanism of DySample can help DM-YOLO achieve high-precision extraction and accurate localization of disease features, which also provides an opportunity for subsequent sampler improvement for YOLOv9.

#### 3.1.2 MPDIoU

Tomato leaf disease symptoms differ in shape and size, and most of the lesion locations overlap to variable extents, making it difficult to extract marginal detail features and localize lesions, which poses a challenge to disease identification. Therefore, the introduction of MPDIoU (Ma and Xu, 2023) enabled the model to focus on overlapping or non-overlapping disease margin areas for the first time, which effectively improved the ability to capture fuzzy marginal features and provides a new idea and tool for solving the above problems.

Based on a rectangle defined with the coordinates of the top left and bottom right points, a minimum distance-based intersection over union, i.e., MPDIoU, was designed, which is able to directly minimize the distance between the predicted bounding box and the ground truth bounding box so as to optimize the accuracy of the bounding box prediction. Its computation is detailed in **Table 1**.

**Table 1:** Computation process of MPDIoU.

Algorithm: Intersection over Union with Minimum Points Distance
**Input:** Two arbitrary convex shapes: $A,B\in S\subseteq R^{n}$ , width and height of input image: w,h
**Output:** MPDIoU
1:For *A* and *B*, $\left(x_{1}^{A},y_{1}^{A}),(x_{2}^{A},y_{2}^{A}\right)$ denote the top-left and bottom-right point coordinates of *A*, respectively, while $\left(x_{1}^{B},y_{1}^{B}),(x_{2}^{A},y_{2}^{A}\right)$ denote the top-left and bottom-right point coordinates of *B*, respectively.
2: $d_{1}^{2}=(x_{1}^{B}-x_{1}^{A})+{\left(y_{1}^{B}-y_{1}^{A}\right)}^{2}$
3: $d_{2}^{2}=(x_{2}^{B}-x_{2}^{B})+{\left(y_{2}^{A}-y_{2}^{A}\right)}^{2}$
4: $MPDIoU=\frac{A\cap B}{A\cup B}-\frac{d_{1}^{2}}{w^{2}+h^{2}}-\frac{d_{2}^{2}}{w^{2}+h^{2}}$

At the model training stage, each predicted box $\beta_{prd}=\left[x^{prd},y^{prd},w^{prd},h^{prd}\right]$ was made as close as possible to the ground truth box $\beta_{gt}=\left[x^{gt},y^{gt},w^{gt},h^{gt}\right]$ by loss function minimization so as to improve the similarity between the predicted box and the ground truth box, as detailed in Equation 6:


(6) $$L=\underset{\Theta }{min}\sum_{\beta_{gt}\in B_{gt}}L(\beta_{gt},\beta_{prd}|\Theta ).$$

In Equation 6, $\beta_{gt}$ denotes a set of ground truth boxes, $\beta_{prd}$ denotes a set of predicted boxes, and Θ is a deep regression model parameter; normally, the norm of $l_{n}$ acts as a typical form of the loss function. However, recent studies have shown that the norm-based loss function does not meet the needs for evaluation metrics, so an MPDIoU-based loss function form was introduced, as shown in Equation 7.


(7) $$L_{MPDIoU}=1-MPDIoU.$$

By optimizing the key point distances between the predicted boxes and the ground truth boxes, MPDIoU obtained rich margin regression information, enhanced the ability to capture the details of disease margins, and improved the localization accuracy of overlapping symptoms. Combined with the dynamic sampling mechanism of DySample, MPDIoU helped DM-YOLO enable high-precision extraction and high-accuracy localization of disease features for real-time detection, which provides a more reliable and efficient solution for actual agricultural disease detection.

## 4 Experimental

### 4.1 Data acquisition

**Figure 4:**
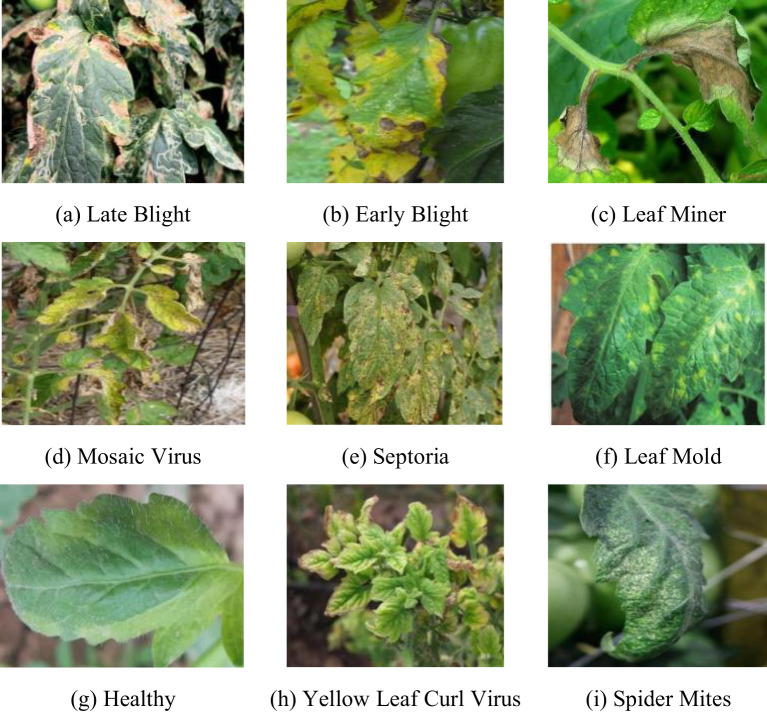
**(A–I)** Healthy leaf and 8 common tomato leaf diseases. Covering **(A)** Late Blight, **(B)** Early Blight, **(C)** Leaf Miner, **(D)** Mosic Virus, **(E)** Septoria, **(F)** Leaf Mold, **(G)** Healthy, **(H)** Yellow Leaf Curl Virus, **(I)** Spider Mlites.

Currently, studies on tomato leaf disease detection focus mainly on lesion identification and localization. However, in actual production, leaf diseases often affect the health status of the whole leaf, varying in morphology and size. Therefore, in this study, the whole leaf was chosen as the detection object to detect diseases from a global perspective. The dataset used in this paper is a tomato leaf disease dataset “Tomato Diseases Detection available on Roboflow platform (Bryan 2023). The dataset consists of images taken in outdoor environments and images captured in laboratory settings. Indoor images are obtained by simulating real environment backgrounds, and outdoor images are taken by researchers under different lighting conditions, such as direct sunlight and leaf occlusion. Additionally, the dataset also includes variations in lesions throughout the disease lifecycle, encompassing a range of sizes, shapes, textures, and colors. As shown in **Figures 4A–I**, the dataset is highly complex and has rich disease diversities, covering 8 common tomato leaf diseases and healthy leaves, including Early light, Healthy, Late light, Leaf Mold, Leaf Miner, Mosaic Virus, Septoria, Spider Mites, and Yellow Leaf Curl Virus.

### 4.2 Data preprocessing

**Figure 5:**
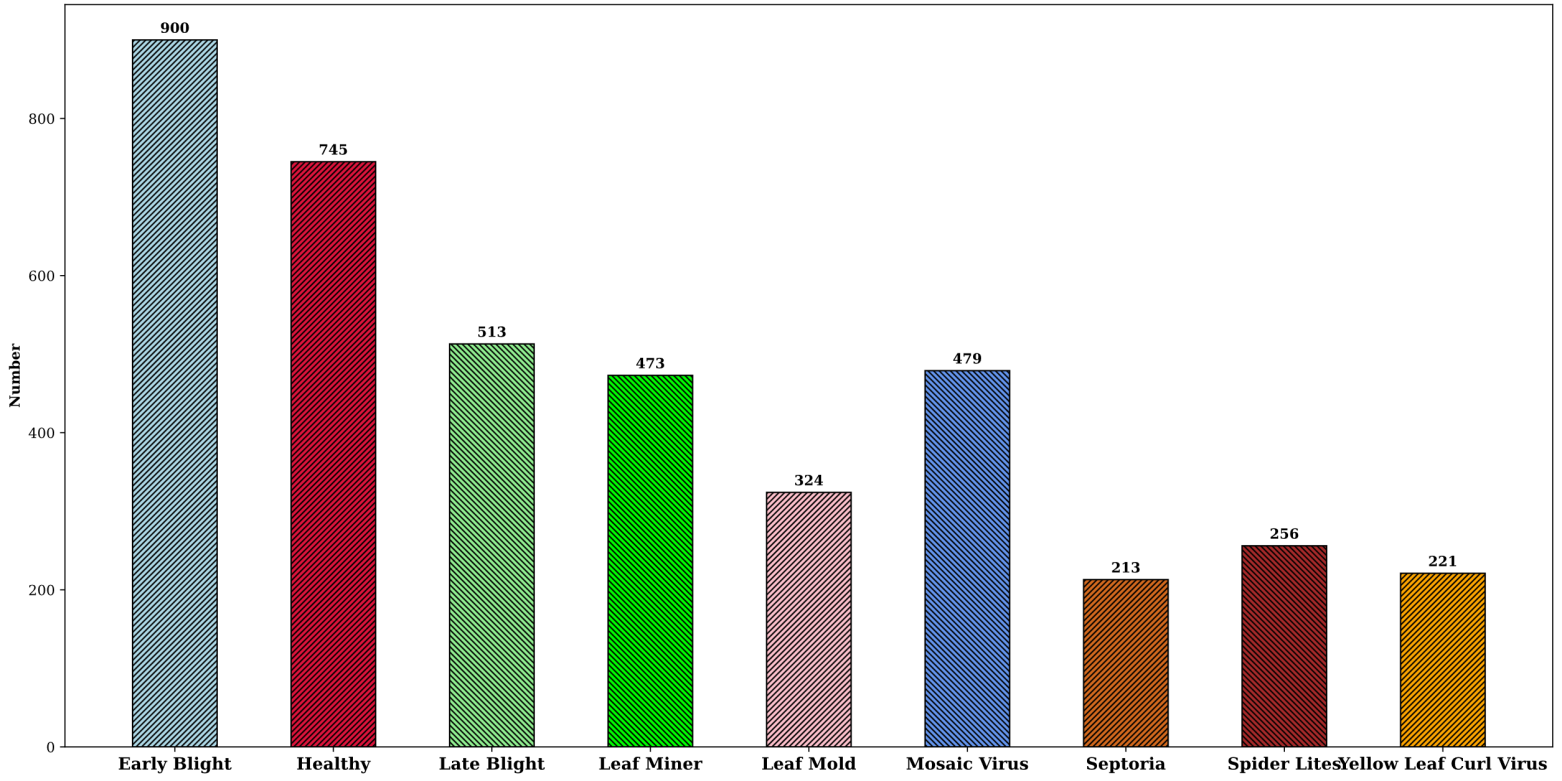
Distribution of disease samples after dataset expansion.

In order to increase the diversity and richness of the training sample images so as to improve the quality and effectiveness of the model training, data augmentation was performed on the original tomato leaf disease dataset, and the augmentation methods include shift, random cropping, rotation, scaling, and brightness control.The dataset was eventually expanded to 4124 images. The data augmentation not only increased the data volume but also significantly improved the robustness and generalizability of the detection model, and **Figure 5** shows the distribution of disease samples after the data expansion.

### 4.3 Dataset splitting

To enhance the effectiveness of model training, we primarily use outdoor images as the training set. However, due to the limited amount of outdoor data, training DM-YOLO effectively is challenging. Therefore, a portion of indoor data is added to expand the dataset. The dataset is structured such that it includes all outdoor data and some indoor data in the training set, with the remaining data allocated to the validation and test sets. By combining both outdoor and indoor images in the training set, we improve the quality of model training while also enhancing the model's robustness, stability, and generalization ability for detecting tomato leaf diseases across diverse environments. As detailed in **Table 2**, the tomato leaf disease dataset is divided into 80% for training, 10% for validation, and 10% for testing.

**Table 2:** Tomato leaf disease dataset distribution.

Dataset	Number of samples
Training set	3300
Test set	412
Validation set	412

### 4.4 Experimental environment

In this study, with YOLOv9 as the baseline model, the DM-YOLO model was constructed to train and evaluate the tomato leaf disease dataset. The experiments in this study were all conducted in the same environment, using training platform NVIDIA A40, 80GB, CUDA 11.3, Ubuntu 20.04, Linux operating system, with PyTorch 1.11.0 as the learning framework and Python 3.8 as the programming language. In the training process, the learning rate was set to 0.01, “batch sizes was set to 16, “epochs was set to 100, and SGD was used as the parameter optimizer. To save computational resource, the training was performed by CUDNN optimization and mixed precision training.

### 4.5 Evaluation metrics

In this paper, metrics P (precision), R (recall), and AP (average precision) are used to measure the detection performance of the model. A value of P represents the ratio of the number of actual leaf disease samples over the number of all detected leaf disease samples, reflecting the ability to identify a relevant object. A value of R focuses on the ratio of the number of correctly detected leaf disease samples over the number of all detected leaf disease samples; the greater the R, the fewer the samples escaping the detection and the better different classes of leaf diseases are detected by the model. AP is the area under the precision–recall curve, which measures the detection performance of the model for a single class of objects. The higher the AP, the better a specific class of diseases are detected. The evaluation metrics are calculated as shown in Equations 8-10, respectively.


(8) $$P=\frac{T_{P}}{T_{P}+F_{P}}\times 100\%$$

(9) $$R=\frac{T_{P}}{T_{P}+F_{N}}\times 100\%$$

(10) $$AP=\int_{0}^{1}Pd(R)\times 100\%$$

where $T_{P}$ denotes the number of samples correctly detected as positive, $F_{P}$ denotes the number of samples falsely detected as positive, and $F_{N}$ denotes the number of samples that are actually positive but falsely detected as negative; the PR curve, with R as the abscissa and P as the ordinate, reflects the precision performance of object detection.

The mean average precision (mAP) is a mean value of the AP values for various classes of diseases, which directly reflects the comprehensive dataset classification ability of the model. The higher the mAP, the better all classes of diseases are detected by the detection model. The calculation of mAP is illustrated in Equation 11


(11) $$mAP=\frac{\sum_{i=1}^{classes}AP_{i}}{classes}\times 100\%$$

where “classes is the number of disease classes. And mAP50 denotes the average accuracy of detecting multiple classes of diseases when the IoU is 0.5.

## 5 Results and analyses

### 5.1 Comparison between samplers

In order to evaluate how different samplers influence the performance of YOLOv9, five different samplers, namely, FADE, SAPA, CARAFE, HWD, and DySample, were introduced into the YOLOv9 model for training and evaluation of the tomato leaf disease detection model versus YOLOv9.

From the experimental results in **Table 3**, it can be seen that different samplers have different degrees of impact on improving the detection performance of the model. Compared with the baseline model, CARAFE improves P and AP by 1.5% and 0.4% respectively, HWD and SAPA improve by 1.2% and 0.7% in terms of P value respectively. It can be seen that DySample has the most outstanding improvement effect, with an improvement of 1.9%, 0.7% and 0.7% in P, AP and mAP50 respectively. It is worth noting that the R and mAP 50 of HWD, FADE, SAPA and CARAFE are slightly lower than those of YOLOv9. The main reason is that the GFLOPs are reduced by 3.1%, 3.7%, 25.3% and 2.2% compared with the baseline model, and the number of parameters is increased by 2.6%, 15.1%, 37.7% and 0.3% respectively compared with the baseline model. It can be seen that the decrease in the detection accuracy of these four samplers is at the expense of the increase in the number of parameters and latency, so it is inevitable to sacrifice some recall and precision. Overall, among these five samplers, DySample performed excellently in metrics R and mAP, as its R and mAP were 1.5% and 2.7% higher than those of CARAFE, respectively, and 1.0% and 2.2% higher than those of HWD, respectively. If the other four samplers are integrated into YOLOv9, not only will the detection accuracy be reduced, but the detection speed and efficiency of the model will be slow, which cannot meet the detection needs of tomato leaf disease in natural environments. The introduction of DySample not only improves the accuracy of the baseline model, but also greatly reduces the number of network parameters and speeds up network inference, which is conducive to ensuring the stability of detection while reducing the structure. These experimental results clearly show that DySample was superior to the other samplers in tomato leaf disease detection, demonstrating its superior detection performance, enabling it to effectively help DM-YOLO fulfill the task of detecting tomato leaf diseases in natural environments, which strongly corroborates the effectiveness and reasonableness of the subsequent improvement using DySample.

**Table 3:** Performance indicators of different sampling methods in detection results.

Samplers	Metrics					
	P (%)	R (%)	AP (%)	mAP50 (%)	Params (M)	GFLOPs (G)
YOLOv9	89.5	**87.4**	93.4	85.0	50716758	234.7
CARAFE	91.0	85.3	93.8	83.5	50897822	239.9
HWD	90.7	84.6	92.2	81.2	52093014	242.4
FADE	86.6	83.0	91.0	79.5	59737654	243.4
SAPA	90.2	83.7	92.5	81.9	81453142	314.0
DySample	**91.4**	86.3	**94.1**	**85.7**	50866913	236.8

87.4.4 value represents the best performance of the R metrics. 94.1 value represents the best performance of the AP metrics. 85.7 value represents the best performance of the mAP metrics. 1.4 value represents the best performance of the P metrics.

CARAFE (Wang et al., 2019) guides the upsampling process with the content of the input features themselves in order to improve the performance of conventional upsampling methods (such as bilinear interpolation and transposed convolution) to generate sharper and more accurate outputs, making it suitable for fine upsampling scenarios such as image super-resolution. HWD (Xu et al., 2023) saves as much information as possible while reducing the spatial resolution of feature maps by wavelet transforms, in order to solve the problems of conventional downsampling methods (such as maximum pooling or strided convolution) in terms of information loss, and better preserve the margin, texture, and detail information of an image. FADE (Lu et al., 2022) selects and enhances data by analyzing the feature distribution of samples, paying special attention to samples that are easy or difficult to classify in the data set. It is suitable for tasks that improve the model's ability to identify complex or confusing samples, such as image classification and target detection. SAPA (Lu et al., 2022) dynamically adjusts the intensity or type of data enhancement and allocates more appropriate data according to the learning state of the model. It is suitable for tasks that require long-term training and gradual enhancement, such as natural language processing. In contrast, DySample (Liu et al., 2023) calculates the differences between the current pixel and the neighboring pixels by differential sampling, and selects only the portions with a greater difference for sampling, so as to improve the sampling rate and efficiency, making it suitable for multi-image processing and computer vision tasks.

**Figure 6:**
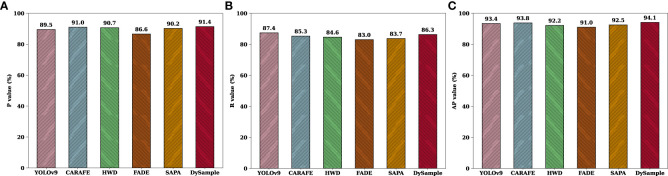
Performance comparison of different samplers.

The comparison results in **Figures 6A–C** show that, the DySample-enhanced model performed well in both detection precision and average precision on the tomato leaf disease dataset, showing excellent detection performance compared to the other two samplers. The main reason is that DySample effectively improved the resolution and information capacity of the disease feature maps through its unique upsampling mechanism, which enabled the model to more accurately extract the key disease features and capture the subtle difference features between similar diseases, and to perform differential sampling, demonstrating its unique role and advantages in accomplishing the tomato disease detection task with DM-YOLO.

### 5.2 Comparison between loss functions

In order to verify the impact of different loss functions on improving the performance of the baseline model, five different loss functions, namely, CIoU, MPDIoU, InnerIoU, InnerCIoU, and InnerMPDIoU, were introduced into YOLOv9 for training and evaluation and compared with YOLOv9.

By analyzing the results in **Table 4**, it can be known that the use of different loss functions had a positive impact on improving the P and AP of the baseline model, as for these five loss functions versus the baseline model, the P values increased by 1%, 1.2%, 1.5%, 0.9%, and 2%, respectively, and the AP values increased by 0.5%, 0.4%, 0.3%, 0.6%, and 0.7%, respectively. In addition, among these five loss functions, MPDIoU performed the best in terms of P, AP, and mAP, in particular, its P value was 1%, 0.8%, 0.5%, and 1.1% higher than those of the other four loss functions, respectively. This is mainly because MPDIoU greatly improved the regression performance of the bounding boxes by minimizing the key point distance between the predicted bounding box and the ground truth bounding box, thereby richer disease information was obtained, making the model more accurate in capturing the leaf margin details and thus more precise in isolation and localization of each overlapping lesion area, significantly improving the overall detection performance of the model.

**Table 4:** Performance indicators of different loss functions.

Models	Metrics			
	P	R	AP	mAP
YOLOv9	89.5	**87.4**	93.4	85.0
YOLOv9-CIoU	90.5	88.5	93.9	84.8
YOLOv9-InnerIoU	90.7	87.5	93.8	85.8
YOLOv9-InnerCIoU	91.0	87.8	93.7	85.3
YOLOv9-InnerMPDIoU	90.4	88.2	94.0	85.2
YOLOv9-MPDIoU	**91.5**	86.5	**94.1**	**86.0**

91.5 value represents the best performance of the P metrics. 87.4 value represents the best performance of the R metrics. 94.1 value represents the best performance of the AP metrics. 86.4 value represents the best performance of the mAP metrics.

CIoU (Zhen et al., 2021) focuses on the position, size, and shape of a box, enabling more comprehensive assessment of the accuracy of the predicted box, making it suitable for scenes requiring precise localization of the object bounding box. InnerIoU (Zhang et al., 2023) pays more attention to the overlapping areas inside a bounding box and is suitable for scenes where attention should be paid to the overlapping areas inside a bounding box. InnerCIoU solves the problem of failing to effectively measure the distance between the predicted box and the ground truth box when both boxes do not overlap, making it suitable for scenes where there is rotation or scaling of the object of interest. InnerMPDIoU takes into account the bounding box overlap, the distance between the center points, and other factors, and is thus suitable for scenes where attention should be paid to the width and height of a bounding box. MPDIoU (Ma and Xu, 2023) pays more attention to the marginal details of the predicted box and is suitable for scenes requiring precise capture of information about the object margins. Different loss functions have different focuses and should be selected depending on specific tasks.

Compared with the other four loss functions, MPDIoU focuses on the overlapping margins between the predicted box and the ground truth box for the first time, enhances the margin detail awareness of the model so as to obtain key features, and has a significant role and unique advantages in YOLOv9, providing the preconditions for subsequent selection of it as an improved loss function.

### 5.3 Comparison between improved models

**Table 5** shows the detection results of the tomato leaf disease dataset by different mainstream improved models. From the analysis of the experimental results in **Table 5**, it can be known that, for each improved model versus the baseline model, P, R, AP, and mAP increased to variable extents. For YOLOv9-Attention-MPDIoU, both R and AP increased by 1.1%. For YOLOv9-GhostConv-MPDIoU, P and AP increased by 1% and 0.5%, respectively. For YOLOv9-DWConv-MPDIoU, P and AP increased by 1.3% and 0.9%, respectively. For YOLOv9-iRMB-MPDIoU, both P and AP increased by 0.8%, mainly due to a fact that cumulative error for the iRMB structure increased with increasing number of network layers, resulting in a slight decrease in accuracy. In contrast, P, AP, and mAP of DM-YOLO increased by 3%, 1.7%, and 1.4%, respectively; compared to the other improved models, DM-YOLO outperformed in P and mAP, with P increasing by 2.2%, 1.7%, 2.3%, 2%, and 1.4%, respectively, and mAP increasing by 3.6%, 0.9%, 1%, 3.8%, and 2.6%, respectively. Overall, the detection performance of YOLOv9 and its improved model is not much different, but it can be clearly seen that as the detection accuracy of other improved models increases, their own network parameters and GFLOPs also increase accordingly. The YOLOv9-ACmix-MPDIoU P and AP increased by 0.9% and 0.2% compared with the baseline model, but this improvement came at the expense of increasing the number of parameters by 5.5% and GFLOPs by 24.3%. Obviously, the trade-off between efficiency and accuracy was not achieved, and the same is true for other samplers. On the contrary, while DM-YOLO increased P, R, and mAP50 by 3%, 1.7%, and 1.4% respectively, its parameter volume and GFLOPs decreased by 1.2% and 2.5%respectively, truly improving detection accuracy. At the same time, the model is lightweight and a trade-off between efficiency and accuracy is achieved. Obviously, among these five improved models, DM-YOLO incorporating DySample and MPDIoU had the best detection performance and was better competent for tomato leaf disease detection tasks.

**Table 5:** Performance indicators of different improved models in detection results.

Models	Metrics					
	P (%)	R (%)	AP (%)	mAP50 (%)	Params (M)	GFLOPs (G)
YOLOv9	89.5	87.4	93.4	85.0	50716758	236.7
YOLOv9-DWConv-MPDIoU	90.8	88.0	94.3	85.5	50644182	233.0
YOLOv9-Attention-MPDIoU	90.2	**88.5**	94.5	85.4	40310428	222.5
YOLOv9-iRMB-MPDIoU	90.3	84.8	94.2	82.8	54415446	339.9
YOLOv9-GhostConv-MPDIoU	90.5	86.7	93.9	83.6	50679894	235.7
YOLOv9-ACmix-MPDIoU	90.4	87.6	93.6	83.8	53687118	312.5
DM-YOLO(Ours)	**92.5**	86.8	**95.1**	**86.4**	50066913	230.8

92.5 value represents the best performance of the P metrics.88.5 value represents the best performance of the R metrics.95.1 value represents the best performance of the P metrics.86.4 value represents the best performance of the mAP metrics.

**Figure 7:**
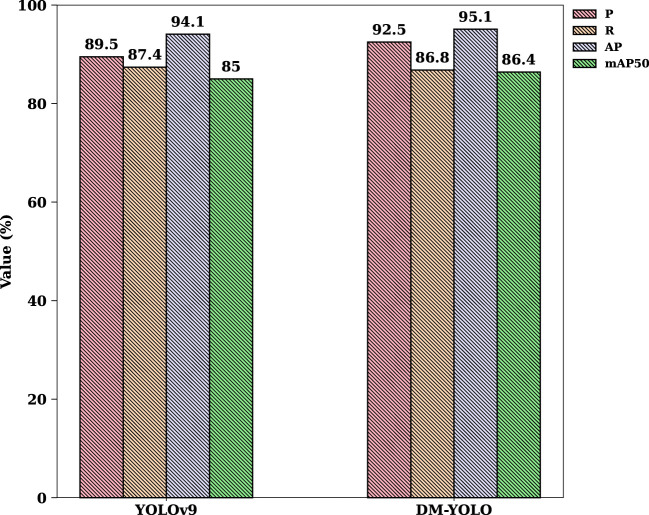
Performance comparison of before and after model improvement.

In order to visualize the improvement effect and verify the feasibility of the improvement, DM-YOLO and YOLOv9 were trained and evaluated on the tomato leaf disease dataset, respectively, and **Figure 7** compares the changes in P, R, AP, and mAP before and after the model improvement.

### 5.4 Comparative experiments with mainstream models

To further verify the detection and generalization capabilities of the proposed DM-YOLO on the tomato leaf disease dataset, this paper first compares 14 other detection models on the same dataset and compares their overall performance with DM-YOLO. The models compared in this paper include YOLOv3, YOLOv5, YOLOv6, YOLOv8, YOLOv10, YOLOv11 (Khanam and Hussain, 2024) and YOLOv9 in different versions to highlight the generalization ability of the proposed model. The experimental results of all the compared models are shown in **Table 6**. Analyzing the experimental results in **Table 6**, it can be seen that compared with the baseline model YOLOv9, although the R value of DM-YOLO is slightly lower, DM-YOLO has improved P, AP, and mAP50 by 2%, 1.7%, and 1.4%, respectively, because a higher recall rate will reduce the accuracy to a certain extent. When considering the network parameters and GFLOPs separately, DM-YOLO is reduced by 1.2% and 14% respectively compared with the baseline model, achieving a trade-off between accuracy and efficiency of tomato leaf disease detection in a natural environment. Compared with other comparison models, DM-YOLO outperforms most detection models in all evaluation indicators, achieves the best balance between precision and recall, achieves the highest mAP value, and can excellently complete the task of detecting tomato leaf diseases in natural environments.

**Table 6:** Performance indicators of different models in the tomato leaf disease dataset.

Models	Metrics			
	P(%)	R(%)	AP(%)	mAP50(%)
YOLOv3	84.1	82.2	88.3	75.4
YOLOv3tiny	77.8	79.8	83.1	59.7
YOLOv5	84.3	81.5	88.9	71.9
YOLOv6	86.5	82.8	89.6	74.3
YOLOv8	88.9	78.7	89.3	74.0
YOLOv9	89.5	**87.4**	93.4	85.0
YOLOv9c	85.9	84.9	91.4	76.8
YOLOv9s	83.2	86.4	91.4	77.1
YOLOv9m	89.6	84.0	91.8	79.2
YOLOv10n	83.3	81.6	89.4	73.4
YOLOv10s	87.4	83.8	91.6	76.2
YOLOv10m	86.7	85.5	91.3	77.5
YOLOv11	82.2	86.0	91.3	77.6
RT-DETR	86.4	83.7	87.2	72.2
LW-DETR	87.9	84.2	90.3	75.4
DM-YOLO(Ours)	**92.5**	86.8	**95.1**	**86.4**

87.4 value represents the best performance of the R metrics.

92.5 value represents the best performance of the P metrics.

95.1 value represents the best performance of the AP metrics.

86.4 value represents the best performance of the mAP50 metrics.

### 5.5 Between-disease comparison in detection results

The tomato leaf disease dataset used in this paper is rich in disease class, and the challenges encountered in detecting lesions in natural environments, such as small lesion size, overlapping lesion areas, and overlapping leaves with each other, lead to insufficient precision in the extraction of lesion sites. Therefore DM-YOLO, a new detection model, is proposed in this paper. To demonstrate the ability of the DM-YOLO to detect different classes of leaf diseases in natural environments, the model was trained and evaluated based on the above training parameters, and the detection results were obtained as shown in **Table 7**. From **Table 7**, it can be seen that DM-YOLO had good overall performance in detecting nine types of tomato leaf diseases. For Early Blight, Healthy, Late Blight, Leaf Miner, Mosaic Virus, Septoria, and Spider Lites in the dataset, all the values of P and AP remained above 90%, and the R and mAP were both higher than 77% and 83%, respectively. In particular, Leaf Miner had the optimal detection results, with P reaching 96.3%, R reaching 98.3%, AP reaching 98.9%, and mAP reaching 91.3%, which satisfied the actual detection requirements. However, for disease classes Leaf Mold and Yellow Leaf Curl Virus, although good performance was made in P and AP, the R and mAP values were not so desirable, and the mAP for Yellow Leaf Curl Virus was only 71%. The main reason is that both diseases resulted in a small lesion area and a low extractability of lesion features, making it not easy to extract small lesion features and localize specific lesions. Therefore, the detection results of Leaf Mold and Yellow Leaf Curl Virus by DM-YOLO are reasonable. Overall, DM-YOLO was able to fulfill the task of detecting most of the common tomato leaf diseases

**Table 7:** Detection results of different diseases under DM-YOLO.

Classes	Metrics			
	P	R	AP	mAP
Helthy	95.2	77.9	94.3	85.7
Septoria	91.8	79.1	91.7	83.3
Leaf Mold	89.5	84.7	94.1	84.1
Late Blight	92.3	89.2	96.9	89.0
Leaf Miner	94.5	**98.3**	**98.9**	**91.3**
Spider Lites	**96.3**	91.2	97.7	90.3
Early Blight	92.1	85.3	93.5	85.0
Mosaic Virus	93.5	88.7	94.1	89.3
Yellow Leaf Curl Virus	87.6	73.3	88.8	71.0
All	92.5	86.8	95.1	86.4

96.4 value represents the best performance of the P metrics.98.3 value represents the best performance of the R metrics.98.3 value represents the best performance of the R metrics.91.3 value represents the best performance of the mAP metrics.

**Figure 8:**
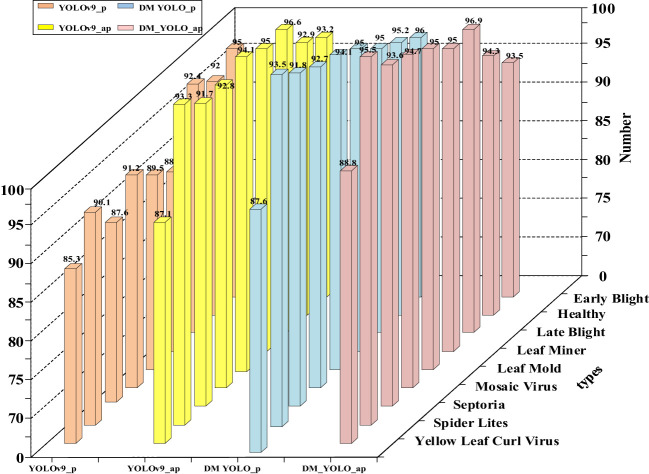
Disease detection performance of before and after model improvement.

In order to better demonstrate the disease detectability of DM-YOLO and facilitate comparison and analysis, the P and AP values of detecting various diseases before and after the model improvement were visualized and compared, as shown in **Figure 8**.

### 5.6 Ablation experiments

To evaluate the effectiveness and feasibility of the DM-YOLO proposed in this paper for tomato leaf disease detection, a number of ablation experiments based on YOLOv9 were conducted. Each individual improvement and a combination of two improvements were added to YOLOv9 and compared with it, aiming to test the effectiveness of each improvement separately so as to elucidate the contribution of each improvement to the overall performance of the model, and the results of the experiments are shown in **Table 8**.

**Table 8:** Results of ablation experiments.

YOLOv9	DySample	MPDIoU	P	R	AP	mAP
√			89.5	**87.4**	93.4	85.0
√	√		91.4	86.3	94.1	85.7
√		√	91.5	86.5	94.1	86.0
√	√	√	**92.5**	86.6	**95.1**	**86.4**

√ indicates adding a module into the baseline model.92.5 value represents the best performance of the P metrics.87.4 value represents the best performance of the R metrics.92.5 value represents the best performance of the AP metrics.92.5 value represents the best performance of the AP metrics.

After incorporating DySample, a lightweight upsampler, into the backbone network, the improved model had improved performance to variable extents in terms of P, AP, and mAP (increasing by 1.9%, 0.7%, and 0.7%, respectively). The results show that DySample could capture the key features of the lesion area more accurately by dynamically adjusting the sampling strategy and could identify the early tiny lesion features quickly and effectively, thus improving the detection accuracy and efficiency of the model.

The introduction of MPDIoU in the detector had a positive impact on improving the model performance, especially in terms of P, AP, and mAP, which increased significantly by 2%, 0.7%, and 1%, respectively, despite a slight decrease in recall. The analysis suggests that by using MPDIoU, the model is able to identify and localize overlapping lesion areas more accurately, better capture lesion margin features, and significantly improve the accuracy of localizing lesion areas.

For a combination of two improved strategies versus the baseline model, the values of P, AP, and mAP increased by 3%, 1.7%, and 1.4%, respectively, indicating significant improvement in model performance. These experimental results strongly indicate the effectiveness of the proposed method. This also just suggests that only by combining both improved methods can we maximize the detection performance and potential of DM-YOLO to fulfill brilliantly the task of tomato leaf disease detection in natural environments.

In conclusion, the authors took advantage of two different improvement strategies to effectively improve the overall detection performance of the model, strongly verified the feasibility of both improvement strategies, and demonstrated that the improved model DM-YOLO is competent for tomato leaf disease detection in complex natural environments, which also provides an effective means of detecting other diseases.

**Figure 9:**
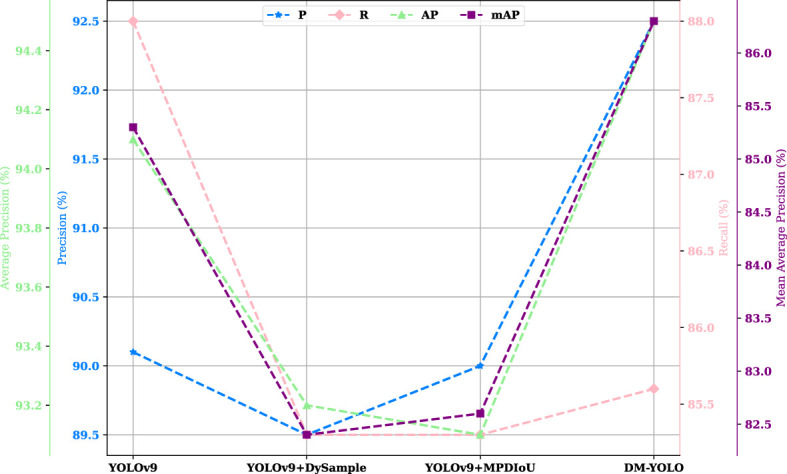
Performance Comparison of ablation experiments.

**Figure 9** visualizes the effectiveness of two improvement strategies. Improving the sampler or the loss function alone had no significant effect on improving the overall performance of the baseline model, while a combination of both improvements resulted in all-around improvement in model performance, with the metrics being 3%, 1.7%, and 1.4% higher than those of the baseline model, respectively, which strongly indicates that the combination of both improvements is conducive to improving the overall detection performance of the DM-YOLO and helping it fulfill a number of detection tasks.

**Figure 10:**
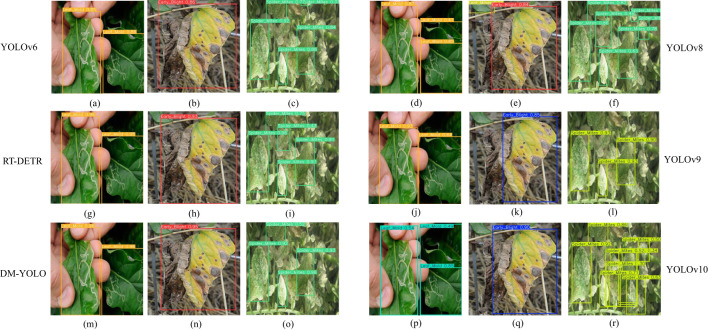
**(A–R)** Comparison of prediction results on tomato disease dataset before and after model improvement.

**Figures 10A–R** shows the visualization of the detection results on the tomato disease dataset before and after the model improvement, with the bright red candidate boxes corresponding to the same class of tomato leaf diseases and the differences in detection precision.

The detection results **Figures 10J, P** in column 1 show that when YOLOv9 and YOLOv10 was disturbed by symptom overlap in natural environments, its detection precision values for Leaf Mold were 93% and 54%, obviously not meeting the detection requirements, in contrast, based on **Figure 10M** in column 1, the precision values of DM-YOLO detecting Leaf Mold reached 95%, enabling precise localization of the margins of Leaf Mold with a small lesion area and enabling high-precision feature extraction, too. The low detection accuracy of the baseline model was mainly due to the difficulty in feature extraction caused by the Leaf Mold, leading to false detection, while the improved model could overcome the interference from the background environment and maintain the accuracy of detection under complex detection environments.

In addition, light variations further affected the extraction of leaf texture features, thereby affecting the accuracy of detection. However, in complex scenes with similar symptoms, such as **Figures 10B, E, K** in column 2, detection accuracy of Early Blight by the baseline model,YOLOv6 and YOLOv8 were 85%,86% and 84% only, in comparison, from **Figure 10N** in column 2, DM-YOLO had a detection precision up to 95%. It is able to learn effectively the marginal features with overlapping similar symptoms and accurately identify and localize precisely Early Blight and Late Blight with similar symptoms. Disease classes differing much in symptoms could be effectively detected by both models. For diseases with small lesion areas and overlapping symptoms, such as early Spider Mlites disease in **Figures 10I, R, L** in Column3, the precision of RT-DETR, YOLOv10, YOLOv9 were 75%, 50% and 90%, while that of DM-YOLO was 93% because it is able to extract precisely the fine marginal features of the small lesions and localize accurately the marginal areas.

The DM-YOLO proposed in this paper is able to suppress the interference from the environment, maintain the robustness and stability of the model detection in complex environments, and keep high precision when facing such factors as symptom overlap, small lesion area, and symptom similarity, making it competent for tomato disease detection tasks in complex natural environments.

## 6 Discussion and limitations

### 6.1 Discussion

Existing YOLO detectors (Zeng et al., 2023; Umar et al., 2024; Liu et al., 2023; Liu and Wang et al., 2021b; Liu et al., 2023) have demonstrated impressive performance on tomato leaf disease datasets. However, detecting tomato leaf diseases in natural environments still faces many challenges, such as light variations, small lesion area, symptom overlap, and leaf occlusion, and existing studies still have limitations in these areas. The DM-YOLO proposed in this paper maintains the same network structure as other YOLO models, which allows for a key improvement to YOLOv9 that is different from the previous ones, i.e., introduction of DySample and MPDIoU, which has strengthened the model's ability to sample leaves with small lesion areas and enhanced the ability to learn details of overlapping margins of disease symptoms, in order to improve the model's ability to extract small lesion features and precision of localizing fuzzy margins while effectively suppressing the interference from natural environments, so as to significantly improve the accuracy of classification and localization by the model. These works not only address the problems in previous studies but also explore new interests of research; nevertheless, more optimized solutions need to be further explored. The authors believe that, given beneficial explorations in addressing the challenges to tomato leaf disease detection in natural environments, DM-YOLO is very promising to provide a powerful technical tool for agricultural disease control and may be a compelling interest of study for future research on object detection in agriculture.

### 6.2 Limitations

Despite remarkable progress made for DM-YOLO on the tomato disease dataset, it still faces a challenge of unbalanced distribution of disease feature samples in the existing dataset. The model proposed in this paper performed poorly in processing images of small lesion samples with similar symptoms and failed to adequately capture the key features of a few classes of lesions, so the precision of identifying and localizing a few classes of diseases needs to be further improved.

## 7 Summary and prospect

In order to improve the accuracy of tomato leaf disease detection in natural environments, the YOLOv model was improved in this paper. Firstly, the lightweight dynamic upsampler DySample was introduced. This improvement made the model more capable and efficient in sampling small area lesions on leaves while effectively reducing the interference from the background environment. Secondly, the loss function was replaced with MPDIoU, which strengthened the model's ability to learn the details of overlapping margins of symptoms and improved the model's ability to capture fuzzy features. The experimental results show that the improved DM-YOLO model was able to accurately recognize tomato leaf diseases in natural environments. Compared with other detection models, DM-YOLO showed excellent detection performance. Significant detection effect was achieved on the public dataset "Tomato Diseases Detection", which further validates its superior generalizability and detection accuracy. Future research work includes: (1) Improvement and optimization of the model structure: Optimize the aggregation of residual blocks (i.e., Multi-Scale Aggregation) to reduce information loss and noise amplification in the process of feature fusion, and enhance the inference speed and detection ability of the model. (2) Improvement of the annotation strategy: Designing a more fine-grained and comprehensive annotation framework (i.e.,rotation labeling strategy), especially for small lesion sample images with similar symptoms, introducing more key information and detailed annotations, such as lesion shapes, edge features, and color changes. (3) Multimodal data fusion: Construct multimodal datasets by combining environmental information (e.g., temperature, humidity, light) and non-visual data such as soil composition during the shooting period. Perform multimodal recognition and fusion to improve the accuracy of tomato leaf disease detection in natural environments. (4) Lightweight network structure design: Examine the components of the model comprehensively from an accuracy-efficiency-driven perspective to reduce redundant structures and improve the detection speed and efficiency of the model.

## Data Availability

The data supporting the conclusions of this article will be made available by the authors, without undue reservation.
